# Challenging the Integrity of Rhythmic Maternal Signals Revealed Gene-Specific Responses in the Fetal Suprachiasmatic Nuclei

**DOI:** 10.3389/fnins.2020.613531

**Published:** 2021-01-07

**Authors:** Vendula Lužná, Pavel Houdek, Karolína Liška, Alena Sumová

**Affiliations:** Laboratory of Biological Rhythms, Institute of Physiology, Czech Academy of Sciences, Prague, Czechia

**Keywords:** circadian clock, development, fetus, maternal entrainment, suprachiasmatic nucleus

## Abstract

During fetal stage, maternal circadian system sets the phase of the developing clock in the suprachiasmatic nuclei (SCN) via complex pathways. We addressed the issue of how impaired maternal signaling due to a disturbed environmental light/dark (LD) cycle affects the fetal SCN. We exposed pregnant Wistar rats to two different challenges – a 6-h phase shift in the LD cycle on gestational day 14, or exposure to constant light (LL) throughout pregnancy – and detected the impact on gene expression profiles in 19-day-old fetuses. The LD phase shift, which changed the maternal SCN into a transient state, caused robust downregulation of expression profiles of clock genes (*Per1*, *Per2*, and *Nr1d1*), clock-controlled (*Dbp*) genes, as well as genes involved in sensing various signals, such as *c-fos* and *Nr3c1*. Removal of the rhythmic maternal signals via exposure of pregnant rats to LL abolished the rhythms in expression of *c-fos* and *Nr3c1* in the fetal SCN. We identified *c-fos* as the gene primarily responsible for sensing rhythmic maternal signals because its expression profile tracked the shifted or arrhythmic maternal SCN clock. Pathways related to the maternal rhythmic behavioral state were likely not involved in driving the *c-fos* expression rhythm. Instead, introduction of a behavioral rhythm to LL-exposed mothers via restricted feeding regime strengthened rhythm in *Vip* expression in the fetal SCN. Our results revealed for the first time that the fetal SCN is highly sensitive in a gene-specific manner to various changes in maternal signaling due to disturbances of environmental cycles related to the modern lifestyle in humans.

## Introduction

In mammalian brain, the paired suprachiasmatic nuclei of the hypothalamus (SCN) harbor principal pacemaker (central clock) (Moore and Eichler, 1972; Ralph et al., 1990) that generates rhythmic signals with a circadian (“approximately a day”) period and orchestrates the phases of oscillators located throughout the body. The prominent role of the SCN among the clocks in the body is determined by 1) its unique structure, comprising a web of mutually interconnected subpopulations of cellular oscillators that ensures the production of a coherent and robust rhythmic signal (Welsh et al., 1995; Herzog et al., 2004; Liu et al., 2007; Webb et al., 2009) and 2) its ability to adjust (entrain) according to the external light/dark cycle, which is achieved via its direct connection with the retina (Zordan et al., 2001; Morin and Allen, 2006). These functions make the SCN a key structure of predictive homeostasis that allows adaptation of physiological and behavioral processes according to expected daily and seasonal changes in external environment.

Previous research has investigated the not yet fully resolved question of when during early life the SCN begin to fulfil their role of the central clock in the body (reviewed in Sumová et al., 2012; Sumová and Čečmanová, 2020). Answering this question is important for understanding the impact of maternal chronodisruption, which may occur in pregnant women exposed to modern lifestyles, on offspring. It has been found that chronodisruption *in utero* leads to pathological phenotypes later in adulthood (Mendez et al., 2016; Richter et al., 2018; Salazar et al., 2018; Varcoe et al., 2018) that may not be rescued by quality of maternal care during postnatal period (Smarr et al., 2017). Therefore, temporal organization during fetal development seems to be important for good health in adulthood.

For experimental purposes, the development of the SCN has been mostly studied in nocturnal rodent species. Morphologically, the rodent SCN develops during the perinatal period in a gradual process, duration of which differs according to the animal species (reviewed in Bedont and Blackshaw, 2015). In rats (a rodent species used in this study), gestation lasts approximately 21–22 days and SCN neurogenesis occurs between the embryonic days 14 (E14) and E17 (Altman and Bayer, 1978). However, synapses start to be formed around E19 and the process is fully completed postnatally (Moore and Bernstein, 1989). At the level of the daily profiles of clock gene expression, which are genes whose protein products are involved in the generation of the circadian signal (reviewed in Lowrey and Takahashi, 2011), the rhythmicity also develops in the SCN gradually. This was demonstrated in the rat SCN examined *in situ* via progressively increasing amplitudes of the rhythmic profiles from the late fetal to the early postnatal stages (Sládek et al., 2004; Kováčiková et al., 2006; Houdek and Sumová, 2014). The developmental process tightly matched the progression of synaptogenesis (reviewed in Bedont and Blackshaw, 2015), which clearly suggests that the robustness of the SCN clock is dependent on its morphological maturation (reviewed in Sumová et al., 2012). Experiments using an *in vitro* approach based on detection of bioluminescence for tracking PER2 protein activity in explanted fetal SCN from mPer2*^*Luc*^* mice confirmed the gradual prenatal development of the SCN clock (Wreschnig et al., 2014; Landgraf et al., 2015; Carmona-Alcocer et al., 2018). The gestational period in mice lasts approximately 19 days and if mouse SCN explants are harvested at E15, synchrony among the oscillators develops spontaneously after several days in culture at the time roughly corresponding to the developmental time *in vivo* (Čečmanová et al., 2019), although other studies were not able to detect this process (Landgraf et al., 2015; Carmona-Alcocer et al., 2018). Intriguingly, in contrast to the adult SCN, the immature SCN is extremely sensitive to culturing procedure itself as well as to nonspecific manipulations of the explants, which can induce robust responses of the fetal SCN clock (Nishide et al., 2008; Čečmanová et al., 2019). Therefore, the *in vitro* model does not seem to fully reflect the *in vivo* situation (Sumová and Čečmanová, 2020), and for studies targeted at investigating the impact of external environment on the fetal SCN, an *in vivo* approach, in which the state of the fetal SCN is assessed *in situ*, is more meaningful. This approach has been used to show that despite the SCN immaturity during the fetal stage, the SCN clock of newborn pups is fully entrained to that of their mother immediately after birth (Davis and Gorski, 1985a,b; Duncan et al., 1986; Reppert and Schwartz, 1986; Weaver and Reppert, 1989; Viswanathan et al., 1994; Bellavía et al., 2006). Additionally, the immature SCN was able to follow changes in the phase of the maternal SCN clock because after exposing pregnant rats to a shift in light/dark (LD) cycle during the late pregnancy, their pups were born with shifted SCN clocks (El-Hennamy et al., 2008). However, applying the shift in the LD cycle closer to delivery term was not effective because the maternal SCN did not have time to fully re-entrain (El-Hennamy et al., 2008). This clearly demonstrates that fetal SCN clock recognizes whether the maternal SCN is fully entrained or whether it is in a state of transition between the original and new LD cycle.

For adaptation to changes in the external LD cycle, the fetal SCN clock, which obviously does not receive information directly from the retina, fully depends on the rhythmic signals sent by the maternal SCN clock. The nature of these signals has previously been extensively studied, and multiple associated pathways have been identified (reviewed in Sumová et al., 2012; Bates and Herzog, 2020; Carmona-Alcocer et al., 2020; Sumová and Čečmanová, 2020). Importantly, the signals are complex and of various origins. They include the rhythmic hormonal pathways (Davis and Mannion, 1988; Viswanathan et al., 1994; Viswanathan and Davis, 1997; Houdek et al., 2015; Čečmanová et al., 2019) as well as pathways related to activity/feeding cycles (Nováková et al., 2010). Apart from their role in setting the proper phase of the fetal clock, the same maternal signals may also be significant for facilitation of fetal clock development. This is supported by our recent finding that glucocorticoid hormones (GCs), which are under control of the maternal SCN and respond to actual arousal state, are important not only for entrainment of the fetal SCN clock but may also facilitate its development (Čečmanová et al., 2019).

Altogether, the results suggest that challenging the integrity and functioning of the maternal circadian system *in vivo* may significantly impact development of the fetal SCN clock. However, knowledge on how this affects the fetal SCN clock at the level of clock and clock-related genes is lacking. To address this issue, we subjected pregnant rats to situations in which production of maternal SCN-driven rhythmic signals to their fetuses was challenged. Specifically, we aimed to manipulate maternal signaling via two different protocols, in which the SCN clock of the pregnant mother was a) in a transient state due to a phase shift in LD cycle or b) arrhythmic due to exposure to constant light (LL). Additionally, to dissect the participation of the behavioral/feeding rhythm as one of the maternal signals, we reimposed the rhythm in the LL-exposed mothers by temporally restricting access to food. Both experimental protocols used in this study were previously employed to demonstrate that in rats, maternal SCN signaling determines the phase of the SCN clock in newborn pups (El-Hennamy et al., 2008) and that rhythmic maternal behavior resets the neonatal SCN (Nováková et al., 2010). In both studies, gene expression profiles were detected in the SCN of newborn pups just after delivery; however, the acute responses within the fetal SCN were not analyzed. Our results revealed that these challenges significantly impacted the fetal SCN in a gene-specific manner, and revealed the high and selective sensitivity of the developing SCN clock to various environmental interventions during pregnancy. The outcome of this study is important because both protocols resemble lifestyle-related challenges to the circadian system that women might be exposed to during pregnancy, such as, jet lag, shift work, an irregular daily regime, or exposure to light pollution at night.

## Materials and Methods

### Animals

Adult male and female Wistar rats (Institute of Physiology, the Czech Academy of Sciences) were housed individually in a temperature-controlled facility at 23 ± 2°C with free access to food and water. All animals were originally maintained under a light/dark cycle with 12 h of light and 12 h of darkness (LD12:12). The time is expressed as Zeitgeber time (ZT); ZT0 corresponds to lights on at 06:00 h and ZT12 corresponds to lights off at 18:00 h. Vaginal smears from females were inspected to determine the estrous cycle phase. On the night of pro-estrus, females were mated with males and on the next morning, they were checked for the presence of sperm in their vaginal smears. In case of sperm positivity, the day was defined as day 0 of embryonic development (E0). Pregnant rats exposed to the experimental protocols described below were monitored for locomotor activity throughout the whole experiment.

All experiments were approved by the Animal Care and Use Committee of the Institute of Physiology and were performed in accordance with the Animal Protection Law of the Czech Republic as well as the European Community Council directives 86/609/EEC. All efforts were made to reduce the suffering of the animals.

### Experimental Protocols

#### Experiment 1 – Effect of the Delay in the LD Cycle on Maternal and Fetal SCN Clocks

The effect of the phase-shift of the maternal signals on the fetal SCN was assessed using pregnant rats (total *n* = 22) that were maintained under the LD12:12 and had unlimited access to food and drinking water during the whole experiment. The pregnant rats were divided into two groups; The control group (“CTRL”) (*n* = 11) remained under the initial LD12:12 regime throughout the whole pregnancy. Body weight (BW) and the amount of food intake were monitored in five females at the times corresponding to E0, E12 and E19. The group assigned as the “Delay” group (*n* = 11) was maintained under the initial LD12:12, but on E14, the LD12:12 cycle was shifted by 6 h in the direction of the phase delay so that the lights were switched off and on 6 h later in the evening and in the next morning, respectively. After the 6-h phase shift, the rats were maintained under this new light/dark regime until E19.

Nonpregnant female rats of the same age were kept under LD12:12 (*n* = 3) for 19 days similar to the experimental pregnant rats. They were weighed and their food consumption was monitored at E0, E12, and E19 for comparison with the pregnant rats.

#### Experiment 2 – Effect of Exposing Pregnant Rats to Constant Light and Restricted Feeding on the Fetuses

To ascertain the effect of maternal feeding regime on the embryonic SCN, two groups of pregnant rats (total *n* = 23) were exposed to constant light (LL) regime from E0 until E19. The LL regime was introduced so that the light was not switched off in the evening on E0. One group of rats was fed *ad libitum* (group “LL-ad lib”) (*n* = 10). In this group, BW and the amount of food were monitored on E0, E14, and E19. The other group was subjected to the restricted feeding (RF) regime (group “LL-RF”) from E0 until E19 (*n* = 13). During the RF regime, food availability was limited to only a 6-h interval from 9:00 to 15:00 (corresponding to ZT3–ZT9 based on the original LD regime). Rats in the LL-RF group were also weighed during the experiment on E0, E14, and E19. Moreover, the food in this group was weighed every day at the beginning and end of the feeding period. Both groups had unlimited access to drinking water during the whole experiment.

Nonpregnant female rats of the same age were kept under LL (*n* = 3) for 19 days, similar to the experimental pregnant rats. They were weighed and their food consumption was monitored on E0, E14, and E19 for comparison with that of the pregnant rats.

### Locomotor Activity Monitoring

For monitoring locomotor activity, the rats were maintained individually in cages equipped with infrared movement detectors attached centrally above the top of each cage. The activity was detected using a circadian activity monitoring system (Dr. Cooper, INSERM, France). The activity was recorded every minute and double-plotted activity records were generated for visualization of the data. The resulting data were analyzed using the ClockLab toolbox (Actimetrics, Illinois, United States).

### Collection of Fetal Samples

For detection of the daily gene expression profiles in the fetal SCN, the pregnant rats in all groups described above were sacrificed at gestational age E19 by decapitation under deep anesthesia by intramuscular injection of a mixture of 150 mg/kg ketamine (Vétoquinol, s.r.o., Czech Republic) and 15 mg/kg xylazine (Bioveta a.s., Czech Republic) at 3 h intervals over a 24 h period (one pregnant rat per time point). Fetuses were sacrificed by rapid decapitation, and at each time point, five fetal heads from each pregnant mother were immediately frozen on dry ice and stored at –80°C for further detection of gene expression using RT-qPCR.

Additionally, for the CTRL, LL-ad lib and LL-RF groups the whole uteruses containing embryos and placentas were extracted and weighed. The bodies without uterus as well as three dyads of embryos and the corresponding placentas from every mother in these three groups were also weighed.

### Detection of mRNA Levels in the Fetal SCN Using RT-qPCR

The fetal heads from all groups in both experiments were sectioned on a cryostat into 20 μm-thick coronal sections containing the medial part of the rostro-caudal extent of the fetal SCN, which was visualized with cresyl violet staining (Sigma-Aldrich, St. Louis, United States). The SCNs were precisely separated bilaterally using a laser microdissector (LMD6000, Leica), as we previously described elsewhere (Houdek and Sumová, 2014). Dissected fetal SCN tissues were collected in a microfuge tube containing RLT buffer from the RNeasy Micro kit (Qiagen, Valencia, United States) and stored until RNA isolation was performed. Total RNA was isolated using the RNeasy Micro kit (Qiagen) according to the manufacturer’s instructions. Isolated RNA samples were immediately reverse-transcribed into cDNA using the HiCapacity cDNA Synthesis Kit (Thermo Fisher, Waltham, MA, United States). The diluted cDNA was then amplified using a LightCycler 480 Real-Time PCR System (Roche, Basel, Switzerland) in 14 μl reactions using 5× HOT FIREPol Probe qPCR Mix Plus (Solis Biodyne, Tartu, Estonia) and TaqMan Gene Expression Assays (Life Technologies, California, United States) for all genes of interest (see Table 1). The ΔΔCt method was used for the quantification of the relative cDNA concentration using mean of three reference genes, namely *Beta-2-Microglobulin* (*B2M, Rn00560865_m1, VIC-labeled*), *Peptidylprolyl Isomerase A* (*Ppia, Rn00690933_m1, VIC*), and *Hydroxymethylbilane Synthase* (*Hmbs*, *Rn01421873_g1, VIC*).

**TABLE 1:** TaqMan probes used to detect the mRNA of genes of interest.

Gene	TaqMan probe
*c-fos*	Rn02396759_m1
*Per1*	Rn01325256_m1
*Per2*	Rn01427704_m1
*Nr1d1*	Rn01460662_m1
*Rorα*	Rn01173769_m1
*Dbp*	Rn01498425_m1
*Vip*	Rn00566449_m1
*Avp*	Rn00566449_m1
*Nr3c1*	Rn00561369_m1

### Statistical Analyses

The t-test was used to compare the behavioral rhythm periods between the experimental groups (CTRL versus Delay groups). One-way ANOVA was used for comparisons of the behavioral rhythm periods between the LL-ad lib and LL-RF groups at two time intervals, and for comparison of maternal BW gain without the uterus and the weights of placentas and embryos among the CTRL, LL-ad lib and LL-RF groups. Two-way ANOVA was used for comparison of the profiles of BW gain and food consumption among the 5 experimental groups (CTRL, LL-ad lib, LL-RF groups, and nonpregnant CTRL and LL groups).

The daily profiles of gene expression were analyzed using 1-way ANOVA (for the detection of the significance of the effect of time) and cosinor analysis (for the detection of significance of the cosinor fit). The cosinor analysis was performed by fitting the data to one of two alternative regression models: either a horizontal straight line (null hypothesis) or a single cosine curve (alternative hypothesis) defined by the equation *Y* = mesor + [amplitude × cos(2 × π × (X-acrophase)/wavelength)] with a constant wavelength of 24 h. The *P* values, coefficient of determination *R*^2^ (goodness of fit), amplitude, acrophase and mesor were determined (see Table 2). The profiles were considered rhythmic when a significant effect of time (confirmed by 1-way ANOVA) and significant cosine fit (assessed by cosinor analysis) were confirmed. Differences between two profiles processed simultaneously in the same plate during RT-qPCR were tested by 2-way ANOVA (see Table 3). All statistics were generated using Prism 7 software (GraphPad, CA, United States).

**TABLE 2:** Results of cosinor analyses.

		CTRL	Delay	LL-ad lib	LL-RF
*c-fos*	acro ± SEM	3.26 ± 1.12	10.66 ± 0.717	–	–
	amp ± SEM	0.592 ± 0.175	0.361 ± 0.064	–	–
	mesor ± SEM	1.564 ± 0.123	0.902 ± 0.047	1.309 ± 0.08	1.078 ± 0.054
	*R*^2^	0.219	0.437	0.070	0.082
	*P*	0.0063	<0.0001	0.0563	0.1745
*Per1*	acro ± SEM	–	–	21.69 ± 1.78	4.86 ± 1.32
	amp ± SEM	–	–	0.119 ± 0.044	0.164 ± 0.062
	mesor ± SEM	0.994 ± 0.078	0.511 ± 0.029	0.923 ± 0.031	1.288 ± 0.042
	*R*^2^	0.029	0.042	0.174	0.149
	*P*	0.5598	0.4125	0.0321	0.0399
*Per2*	acro ± SEM	5.13 ± 1.18	10.11 ± 1.00	–	–
	amp ± SEM	0.383 ± 0.136	0.207 ± 0.053	–	–
	mesor ± SEM	1.709 ± 0.09	0.828 ± 0.038	0.718 ± 0.0186	0.72 ± 0.021
	*R*^2^	0.166	0.280	0.131	0.052
	*P*	0.0268	0.0014	0.0021	0.3379
*Nr1d1*	acro ± SEM	–	12.16 ± 1.30	18.35 ± 0.99	1.34 ± 1.04
	amp ± SEM	–	0.12 ± 0.039	0.444 ± 0.092	0.333 ± 0.084
	mesor ± SEM	1.22 ± 0.065	0.67 ± 0.028	1.588 ± 0.055	1.666 ± 0.062
	*R*^2^	0.012	0.196	0.195	0.285
	*P*	0.7907	0.0129	<0.0001	0.0012
*Rorα*	acro ± SEM	11.94 ± 1.36	13.35 ± 1.45	–	2.89 ± 0.96
	amp ± SEM	0.163 ± 0.055	0.137 ± 0.049	–	0.16 ± 0.041
	mesor ± SEM	0.905 ± 0.04	0.769 ± 0.039	1.005 ± 0.043	1.037 ± 0.029
	*R*^2^	0.199	0.160	0.089	0.277
	*P*	0.0184	0.0281	0.0435	0.0015
*Dbp*	acro ± SEM	22.95 ± 1.45	–	–	0.84 ± 1.43
	amp ± SEM	0.315 ± 0.117	–	–	0.152 ± 0.053
	mesor ± SEM	1.054 ± 0.084	0.537 ± 0.023	1.22 ± 0.041	1.125 ± 0.039
	*R*^2^	0.162	0.078	0.142	0.170
	*P*	0.0352	0.1886	0.0012	0.0221
*Vip*	acro ± SEM	16.18 ± 1.30	16.07 ± 1.16	14.87 ± 1.29	16.34 ± 0.67
	amp ± SEM	0.301 ± 0.109	0.271 ± 0.089	0.371 ± 0.093	0.658 ± 0.125
	mesor ± SEM	1.128 ± 0.075	0.968 ± 0.061	1.078 ± 0.072	1.724 ± 0.085
	*R*^2^	0.155	0.186	0.226	0.405
	*P*	0.0293	0.0147	0.001	<0.0001
*Avp*	acro ± SEM	15.41 ± 0.81	16.36 ± 0.99	17.78 ± 0.71	20.57 ± 1.21
	amp ± SEM	0.323 ± 0.071	0.386 ± 0.105	0.452 ± 0.096	0.367 ± 0.114
	mesor ± SEM	0.731 ± 0.05	0.769 ± 0.072	1.147 ± 0.071	1.166 ± 0.081
	*R*^2^	0.342	0.255	0.325	0.214
	*P*	0.0002	0.0028	0.0001	0.0091
*Nr3c1*	acro ± SEM	21.39 ± 1.01	–	20.45 ± 1.30	20.66 ± 1.33
	amp ± SEM	0.235 ± 0.063		0.244 ± 0.087	0.203 ± 0.07
	mesor ± SEM	1.026 ± 0.044	0.676 ± 0.034	1.44 ± 0.059	1.377 ± 0.05
	*R*^2^	0.264	0.030	0.186	0.172
	*P*	0.0022	0.524	0.022	0.0211

*The data show mean and SEM for acrophases (acro), amplitudes (amp), mesors (mesor), goodness of fit *R*^2^ and significance levels (*P*; gray) of the daily gene expression profiles detected in the SCN of fetuses from mothers in the control group (CTRL) and the groups exposed to 6 h phase delay in LD12:12 (Delay), constant light with *ad libitum* feeding (LL-ad lib) and constant light with restricted feeding regime (LL-RF). The profiles are shown in the Figures 2, 5.*

**TABLE 3:** Results of 2-way ANOVA analyses.

		CTRL vs delay		LL-ad lib vs LL-RF	
Gene	Two-way ANOVA	*P* value	*P* value summary	*P* value	*P* value summary
*c-fos*	Interaction	<0.0001	****	0.0022	**
	Time	<0.0001	****	0.0012	**
	Group	<0.0001	****	<0.0001	****
*Per1*	Interaction	0.0692	ns	0.0572	ns
	Time	0.4003	ns	0.1254	ns
	Group	<0.0001	****	<0.0001	****
*Per2*	Interaction	0.3168	ns	<0.0001	****
	Time	0.0205	*	<0.0001	****
	Group	<0.0001	****	0.0609	ns
*Nr1d1*	Interaction	0.1673	ns	<0.0001	****
	Time	0.8828	ns	0.0008	***
	Group	<0.0001	****	0.7785	ns
*Rorα*	Interaction	0.8877	ns	<0.0001	****
	Time	0.0072	**	0.0003	***
	Group	0.0226	*	0.4578	ns
*Dbp*	Interaction	0.4036	ns	0.0004	***
	Time	0.0666	ns	0.0002	***
	Group	<0.0001	****	0.0205	*
*VIP*	Interaction	0.0024	**	0.1359	ns
	Time	0.0006	***	<0.0001	****
	Group	0.0489	*	<0.0001	****
*AVP*	Interaction	0.0509	ns	0.0043	**
	Time	<0.0001	****	<0.0001	****
	Group	0.5173	ns	0.3543	ns
*Nr3c1*	Interaction	0.0488	*	0.6803	ns
	Time	0.2163	ns	0.0104	*
	Group	<0.0001	****	0.3721	ns

*Comparison between the gene expression profiles in the fetal SCN of the control group (CTRL) and the group exposed to 6-h phase delay in LD12:12 (Delay), and between the group exposed to constant light with *ad libitum* feeding (LL-ad lib) and constant light with restricted feeding regime (LL-RF). The gene expression profiles are shown in Figures 2, 5. The results of significance levels (P) for the interaction between groups, the effect of time and the effect of group (gray) are shown.*

## Results

### Experiment 1 – Effect of the Delay in the LD Cycle on the Maternal and Fetal SCN Clocks

#### The Maternal SCN-Driven Locomotor Activity Rhythm Adjusts Gradually to the 6-h Phase Delay of the LD Regime

**FIGURE 1:**
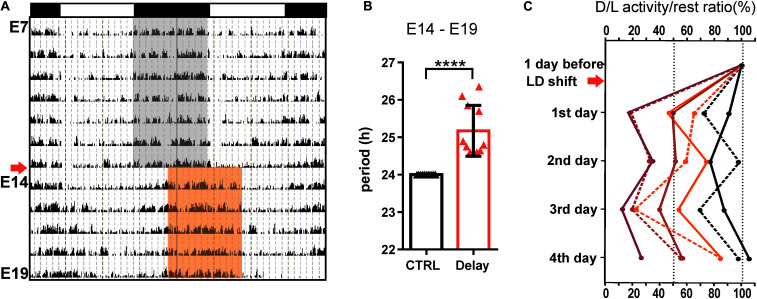
Effect of a 6-h delay in the light/dark cycle on the maternal locomotor activity rhythm. **(A)** Representative double-plotted actogram of a pregnant Wistar rat demonstrating its locomotor activity from embryonic day (E) 7 until E19. The rats were maintained in the original light/dark cycle LD12:12 (as shown in the upper x-axis; the gray area corresponds to darkness), and they were exposed to a 6-h delay in the LD cycle (arrow) by delaying the switch to lights off on E14 (darkness in the new LD cycle is shown as the red area). The fetuses were sampled on E19. **(B)** Circadian periods of the locomotor activity rhythms of pregnant rats determined for the interval between E14 and E19. The rats maintained in the original LD12:12 cycle were assigned as the control group (CTRL; in black; *n* = 11), and those exposed to the 6-h delay in the LD cycle were assigned as the Delay group (Delay; in red; *n* = 11). Data are expressed as the mean ± SD; *t*-test ****P < 0.0001. **(C)** Percent changes in activity/rest ratios of individual pregnant rats calculated as the amount of activity during the actual dark and light phases of each day. The 1st day after the shift corresponds to E15 and the 4th day corresponds to E18. The pregnant rats were sacrificed at specific times on E19. For each pregnant rat, the activity/rest ratio before the LD shift was determined as 100%.

To expose the fetuses to a shift in maternal rhythmic signals, we delayed the LD cycle by 6 h starting on E14 and the pregnant rats were maintained in the new LD regime for the next 5 days (Delay group; *n* = 11). The actual state of the maternal SCN clock was assessed based on the adaptation of their locomotor activity rhythm to the new LD regime. The pregnant rats were fully entrained to the original LD cycle (period tau = 24 h) and after the phase shift, the activity onset and offset started to gradually become delayed via transient cycles (as shown in the representative double-plotted actogram in Figure 1A) with a period tau > 24 h (Figure 1B). The mean period was 25.17 ± 0.2 (mean ± SD), which means that the SCN clock was delayed by approximately 1 h a day. The effectiveness of the achievement of the steady-state aligned with the new LD cycle was variable among the pregnant rats, as assessed by calculating the activity/rest ratios for each rat (*n* = 7) before the shift (the amount of activity during the dark versus the light phase of the actual LD cycle was set as 100%) and then on each of the 4 days following the shift (Figure 1C). The 5th day was not included in the calculation because on that day (E19), the sampling of fetuses started; thus, for some pregnant rats, the full day record was missing. By the 4th day after the shift, out of the total of seven monitored pregnant rats, two fully adapted (ratio of approx. 100%; black lines) and two almost fully adapted (ratio of approx. 85%; orange lines) to the new LD regime; however, the activity of three of the rats was not entrained according to the new LD cycle (ratios approx. 25–60%; red lines). These results show that before E19, the SCN clock appeared to be in a transient state in all of the pregnant rats and full adaptation to the new LD cycle was achieved in some of them only shortly before sampling. Therefore, during the last 5 days of embryogenesis, the fetal SCN clocks of the Delay group developed under nonstable rhythmic signals from the maternal SCN.

#### The Delay in the LD Cycle Dampens the Amplitudes of the Gene Expression Profiles and Shifts the *c-fos* Expression Profile in the Fetal SCN

**FIGURE 2:**
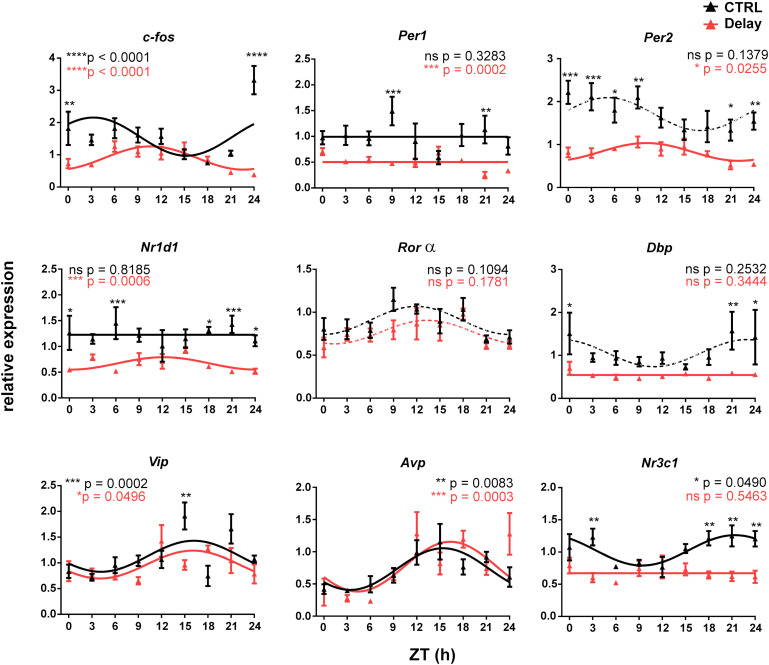
Effect of a 6-h delay in the light/dark cycle on gene expression profiles in the fetal SCN. Pregnant Wistar rats that were maintained under the original LD12:12 cycle (CTRL group; *n* = 11) or exposed to a 6-h delay in the LD cycle (Delay group; *n* = 11) were sacrificed on E19 in 3-h intervals over 24 h period. The fetal SCN of the CTRL group (black lines and black triangles) and the Delay group (red lines and red triangles) were dissected and processed by RT-qPCR to detect the daily profiles of the relative expression of selected genes (*c-fos*, *Per1*, *Per2*, *Nr1d1*, *Rorα, Dbp*, *Vip*, *Avp*, and *Nr3c1*). Time is expressed as Zeitgeber time (ZT); ZT0 corresponds to lights on and ZT12 corresponds to lights off based on the original LD cycle. Data are expressed as the mean ± SEM; each time point corresponds to 4–5 embryos from one mother. The data were fitted with cosine curves (for the results, see Table 2) and analyzed by 1-way ANOVA (*P* values shown in color corresponding to each group are depicted in the upper parts of each graph); solid cosine curve means significant result of both cosinor analysis and 1-way ANOVA, dashed cosine curve means significant result of cosinor analysis but not of 1-way ANOVA, and straight line means nonsignificant result of cosinor analysis. Finally, the differences between the profiles were tested by 2-way ANOVA (results are shown in Table 3; the stars above the time points depict the time when the values significantly differed).

We assessed the impact of the transient state of the maternal SCN clock on the fetal clock by comparing the gene expression profiles within the fetal SCN between the CTRL group (no shift) and the Delay group (shift in the LD cycle as described above) (Figure 2). We selected genes that we believed might potentially respond to altered maternal cues based on our studies and previous studies by others, namely, the immediate early gene *c-fos*, clock genes (*Per1*, *Per2*, *Nr1d1*, and *Rorα*), the clock-controlled gene *Dbp*, genes coding neurotransmitters (*Vip* and *Avp*) and the GC receptor (*Nr3c1*).

The gene expression rhythms in the fetal SCN on E19 are typically shallow, and therefore, we considered the presence of circadian rhythms based on the combination of criteria as described in the section “Materials and Methods,” for which a significant cosinor fit and a significant effect of time by 1-way ANOVA were required (for the results of the cosinor analysis, see Table 2; for the results of 1-way ANOVA, see the significance in the graph inserts in Figure 2). Based on these criteria, in the CTRL group, the expression profiles of the clock genes *Per1*, *Per2*, *Nr1d1*, and *Rorα*, and the clock-controlled gene *Dbp* were arrhythmic because they did not meet both requirements for significance. In contrast, expression of *c-fos*, *Vip*, *Avp*, and *Nr3c1* exhibited shallow but significant circadian rhythms. The difference between the CTRL and Delay groups was further assessed by 2-way ANOVA (Table 3; the significant differences revealed by the post hoc analyses are depicted as stars at the corresponding time points in the individual graphs of Figure 2). Most of the expression profiles of the Delay group were significantly dampened (for all genes with the exception of *Vip, Avp*, and *Rorα*) as shown by the decrease in their mesors (Table 2), which was independent of whether these profiles were rhythmic in the CTRL group or not. The expression of *Per2* was one of the most robustly suppressed. Therefore, disrupting the rhythmic signaling due to transient state of the maternal SCN significantly suppressed expression of clock genes and clock-related genes in the fetal SCN but, interestingly, had no effect on the expression of genes encoding SCN neurotransmitters. Furthermore, the results reveal the recognizable role of *c-fos* in sensing the maternal signals within the fetal SCN because it was the only rhythmically expressed gene that shifted according to the maternal SCN (effect on the group by 2-way ANOVA: *P* < 0.0001). Additionally, the plausible role of GCs in maternal signaling is supported by the fact that the rhythm in expression of the GC receptor (*Nr3c1*) was abolished in the Delay group.

Altogether, the results of Experiment 1 clearly demonstrate that rhythmic maternal signals facilitate gene expression in the fetal SCN because their disruption due to the transitional state of the maternal SCN attenuates the expression levels. Additionally, the data suggest that maternal entrainment of the fetal clock is achieved by signaling pathways employing *c-fos* and GCs.

### Experiment 2 – Effect of Exposing Pregnant Rats to Constant Light and Restricted Feeding on Fetuses

#### LL and RF Differently Affect the Body Weight, Food Consumption, and Weight of the Placentas and Fetuses of Pregnant Wistar Rat, but Not the Size of the Litter

**FIGURE 3:**
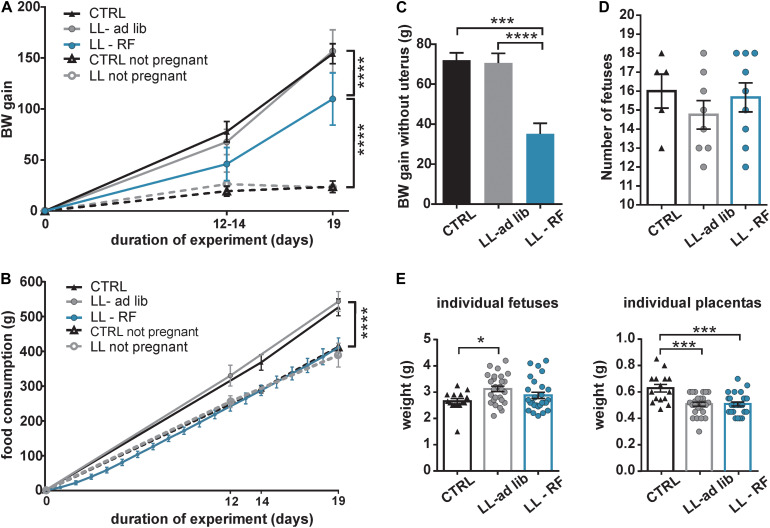
Effect of the exposure of pregnant rats to constant light and a restricted feeding regime on the body weight, food intake, litter size and weight of placenta and fetuses. **(A)** Comparison of the body weight (BW) gain of five groups of Wistar rats over 19 days. Control pregnant (CTRL; *n* = 5) and nonpregnant (CTRL-nonpregnant; *n* = 3) rats were fed *ad libitum* and maintained under LD12:12. Additionally, pregnant (LL-ad lib; *n* = 8) and nonpregnant (LL-nonpregnant; *n* = 3) rats were exposed to constant light and fed *ad libitum*. Finally, one group of pregnant rats maintained under LL was exposed to restricted access to food (LL-RF; *n* = 10). The rats were weighed at the beginning of pregnancy (E0), on E12–E14 and at the end of the experiment on E19 or at the corresponding time intervals for the nonpregnant rats. Data are the mean ± SD. The values of BW gain after 19 days were compared between the groups by 2-way ANOVA ****P < 0.0001). **(B)** Comparison of food consumption (weight of the pellets in grams) between the five groups of rats described in **(A)**. Data are mean ± SD. The weights of consumed food after 19 days were compared by 2-way ANOVA (****P < 0.0001). **(C)** The pregnant rats in the three experimental groups described in **(A)** (CTRL, LL-ad lib, and LL-RF) were sacrificed at E19 and their bodies were weighed after the whole uterus containing embryos and placentas was removed. The weights of the separated fetuses and placentas are shown in **(E)**. For each rat, the value of BW gain was calculated relative to BW at E0. Data are the mean ± SEM. The values were compared between the groups by 1-way ANOVA (****P < 0.0001; ***P = 0.0004). **(D)** Number of fetuses (mean ± SEM) from each mother of the three groups described in **(A)** (CTRL, LL-ad lib, and LL-RF). The data were compared by 1-way ANOVA. **(E)** Weights of individual fetuses and placentas (mean ± SEM) of three groups of mothers as described in **(A)** (CTRL, LL-ad lib, and LL-RF). Three fetuses and placentas from each pregnant rat were measured. The data were compared by 1-way ANOVA (***P_*CTRL vs LL–ad lib*_ = 0.001; *P*_*CTRL vs LL–RF*_ = 0.002; *P = 0.025).

We tested the effect of the attenuation of rhythmic signals derived from the maternal SCN on fetuses by exposing the pregnant rats to LL (LL-ad lib group). Additionally, another group of pregnant rats maintained under LL was exposed to RF (LL-RF group), which provided the fetuses with rhythmic signals derived from the maternal feeding/activity rhythm. Because the experimental protocol involved the manipulation of food access, we first tested whether it impacted BW gain and the weight of the placentas and embryos of the pregnant rats. The pregnant rats from CTRL (*n* = 5), LL-ad lib (*n* = 10), and LL-RF (*n* = 13) groups were weighed on E0, E12–14, and E19 together with a group of age-matched nonpregnant rats maintained under LD12:12 and LL (*n* = 3 in both groups). The BW gain at the time corresponding to E19 was compared between the groups (Figure 3A). As expected, the pregnant rats in LL-RF group gained less weight than those in the CTRL and LL-ad lib groups (*P* < 0.0001). Interestingly, exposure to the LL regime without restricting food access had no effect on BW gain because there were no significant differences between CTRL and LL-ad lib groups (*P* = 0.9973) or between the nonpregnant rats maintained on LD cycle versus LL (*P* > 0.9999) (Figure 3A). To assess whether the effect of RF on BW was due to a change in the amount of consumed food, we monitored the food consumption of pregnant rats from the LL-RF group by weighting the pellets every day of the RF protocol. In all other groups (CTRL, LL-ad lib and both nonpregnant groups), food consumption was determined on E0, E12–14, and E19, or at the corresponding times for the nonpregnant rats (Figure 3B). In accordance with the BW gain, there were no significant differences in amount of food consumed between the CTRL and LL-ad lib groups (*P* > 0.9999) or between both nonpregnant groups (*P* = 0.9939) at the end of the experiment. The pregnant rats from the LL-RF group ate less food than those from the LL-ad lib and CTRL groups (for both groups: *P* < 0.0001), but the amount of food was comparable to that consumed by both nonpregnant groups (*P*_*LL–RF vs CTRL non*__*pregnant*_ > 0.9999 and *P*_*LL–RF vs LL non*__*pregnant*_ = 0.6142) (Figure 3B). The lower BW gain in the LL-RF group compared to that in the LL-ad lib and CTRL groups was due to the lower BW gain of the maternal body itself after uterus and embryo removal (Figure 3C). The litter sizes were comparable between the CTRL, LL-RF, and LL-ad lib groups (*P*_*LL–RF vs LL–ad lib*_ = 0.6657; *P*_*LL–RF vs CTRL*_ = 0.9592; *P*_*CTRL vs LL–ad lib*_ = 0.5796) (Figure 3D).

Although RF decreased the BW gain due to reduced food intake in the pregnant rats of the LL-RF group, they were apparently able to compensate for it to maintain the same placenta and embryo weights as the LL-ad lib group (*P*_*LL–ad lib vs LL–RF groups*_ = 0.9978 and 0.2231 for placentas and embryos, respectively) (Figure 3E). Importantly, LL exposure on its own significantly reduced the weights of the placentas, which were lower in both LL-exposed groups compared with those in the CTRL group (*P*_*CTRL vs LL–ad lib*_ = 0.0001 and *P*_*CTRL vs LL–RF*_ = 0.0002). However, the weights of the embryos were slightly higher in both LL-exposed groups, although a significant difference was confirmed only between CTRL and LL-ad lib groups (*P* = 0.0247). Therefore, although the LL-ad lib group gained the same amount of BW as the CTRL group during pregnancy, their embryos were slightly heavier and the placentas were less well developed.

#### The Locomotor Activity Rhythm of Pregnant Rats Progressively Attenuates Due to Exposure to LL and Is Re-established by Concurrent Exposure to RF

**FIGURE 4:**
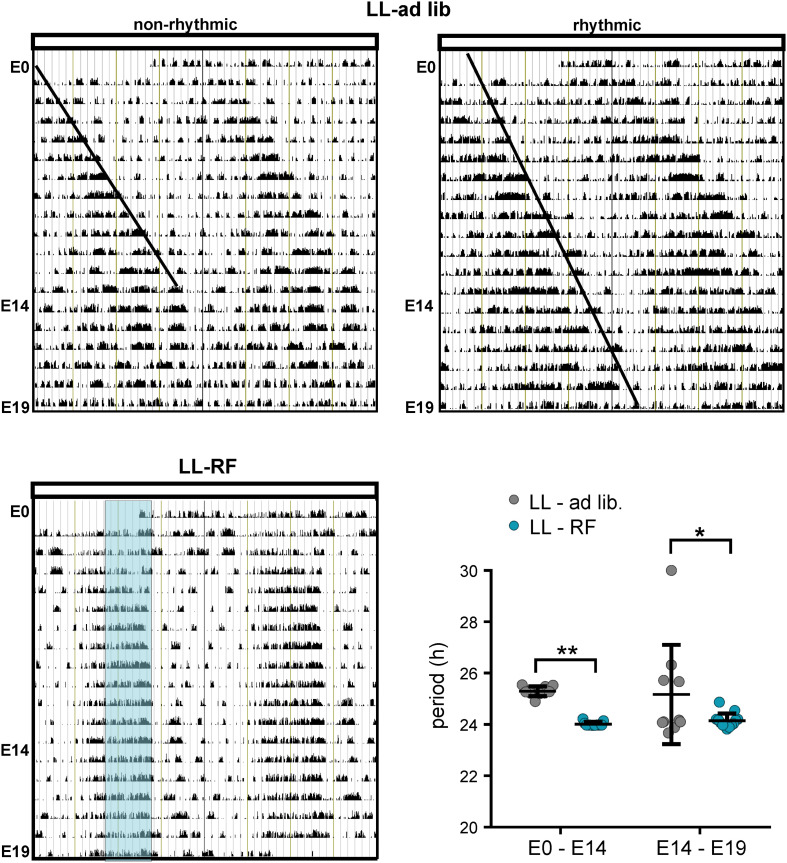
Effect of constant light and the restricted feeding protocol on the locomotor activity of pregnant rats. **(A)** Representative double-plotted actograms of two pregnant Wistar rats kept under constant light (LL) and fed *ad libitum* during entire experiment (E0–E19) (LL-ad lib). The actogram on the left side shows an example of the activity observed in most of the pregnant rats, which became completely nonrhythmic starting before E19. The actogram on the right side is an example from one pregnant rat, which was the exception and remained rhythmic throughout the measurement (the fetal SCNs of this rat were excluded from the experiment). The lines drawn in the actograms are eye-fits of the activity offsets. **(B)** Representative double-plotted actogram of a rat exposed to LL along with the restriction of access to food for only 6 h (LL-RF group). The blue area represents the feeding time (local time 9:00–15:00). The activity of all pregnant rats in the group was adjusted according to the food availability. **(C)** Circadian periods of the locomotor activity rhythms in the LL-ad lib (*n* = 10) and LL-RF (*n* = 13) groups during the intervals E0–E14 and E14–E19. Data are expressed as individual values and means ± SD. Data were compared between both groups by 2-way ANOVA (**p = 0.0035; *p = 0.0211).

The locomotor activity was monitored in pregnant rats of the LL-ad lib (*n* = 10) and LL-RF (*n* = 13) groups from E0 until E19 (representative actograms are shown in Figures 4A,B). Exposure to LL with *ad libitum* feeding caused immediate lengthening of the circadian periods of the locomotor activity rhythms in all mothers (Figure 4A); the mean period between E0 and E14 was 25.30 ± 0.38 h (Figure 4C). Thereafter, during the last 5 days of pregnancy, the long period-rhythms gradually weakened (see the higher variability of the estimated periods in the interval between E14 and E19 in Figure 4C). Although the dynamics of the rhythm weakening were variable among the pregnant rats, all of the mothers but one became completely arrhythmic by E19, which is when the fetuses were collected. The data obtained from the single mother that remained rhythmic throughout pregnancy (shown in Figure 4A; actogram on the right side assigned as “rhythmic”) were excluded from the study. In the pregnant rats of the LL-RF group, the activity rhythm also started to exhibit a long period up to the 5th day after exposure to LL, but then the rats reorganized their activity patterns and synchronized themselves with the time of food availability (the mean period was 24.02 and 24.14 for the E0–E14 and E14–E19 intervals, respectively; Figure 4C). This indicates that the pregnant rats started to be active in the expectation of food (food anticipatory activity) and ceased their activity immediately after food was removed from their cages (Figure 4B). Therefore, whereas in the LL-ad lib group, the SCN-driven locomotor activity rhythm was attenuated/absent during the last 5 days before the sampling of the fetuses, in the LL-RF group, the rhythm was reinforced by food availability.

#### Exposure of Pregnant Rats to LL and LL + RF Selectively Influences Expression of Genes in the Fetal SCN

**FIGURE 5:**
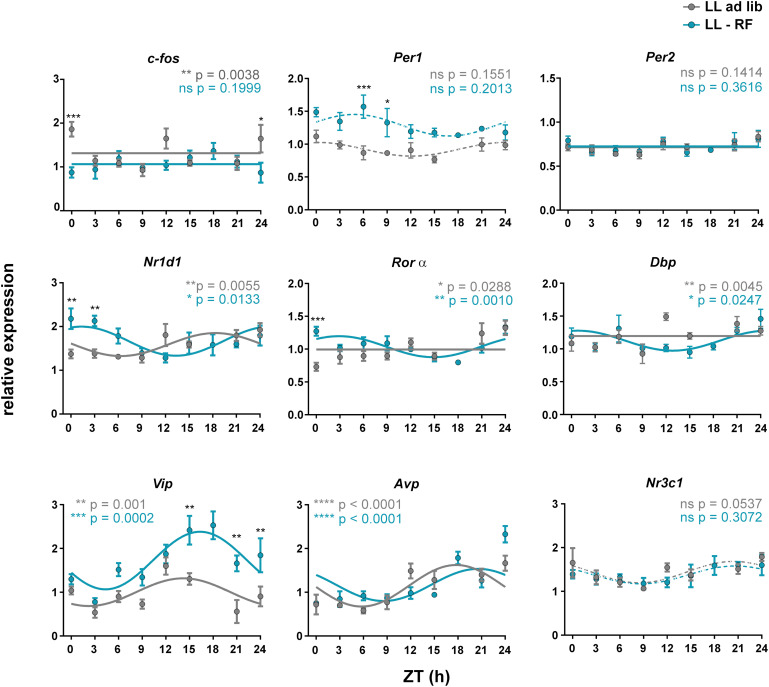
Effect of constant light and the restricted feeding protocol on gene expression profiles in the fetal SCN. Daily profiles of the relative mRNA expression of selected genes (*c-fos*, *Per1*, *Per2*, *Nr1d1*, *Rorα, Dbp*, *Vip*, *Avp*, and *Nr3c1*) in the SCN of 19-day-old fetuses collected from pregnant rats maintained under constant light and either fed *ad libitum* (LL-ad lib group; gray lines and gray circles) or exposed to a restricted feeding regime (LL-RF group; blue lines and blue circles). The pregnant rats were sacrificed in 3 h intervals over 24 h. Time is expressed as Zeitgeber time (ZT); ZT0 corresponds to lights on and ZT12 corresponds to lights off based on the original LD cycle. Data are expressed as the mean ± SEM; each time point corresponds to 4–5 embryos from one mother. The data were fitted with cosine curves (for the results, see Table 2) and analyzed by 1-way ANOVA (*P* values shown in color corresponding to each group are depicted in the upper parts of each graph); solid cosine curve means significant result of both cosinor analysis and 1-way ANOVA, dashed cosine curve means significant result of cosinor analysis but not of 1-way ANOVA, and straight line means nonsignificant result of cosinor analysis. Finally, the differences between the profiles were tested by 2-way ANOVA (the results are shown in Table 3; the stars above the time points depict the time when the values significantly differed).

We examined whether the LL-induced attenuation of maternal SCN-derived signals without (LL-ad lib group) and with (LL-RF group) imposed activity/feeding rhythms affected gene expression in the SCN of 19-day-old fetuses. The daily expression profiles of the same genes as those examined in Experiment 1 were analyzed (Figure 5). In the LL-ad lib group, the expression profiles of most genes did not meet the criteria for rhythmicity (significant cosinor fit and effect of time by 1-way ANOVA), namely the profiles of *c-fos*, *Per1*, *Per2*, *Rorα*, *Dbp*, and *Nr3c1*, although expression profile of *Nr1d1* exhibited a shallow rhythm (for the cosinor analysis data, see Table 2; the 1-way ANOVA results are depicted in the graph inserts of the Figure 5). Based on the comparison with the profiles that were rhythmic in the CTRL group in Experiment 1 (Figure 2) it was obvious that LL exposure abolished the rhythms in *c-fos* and *Nr3c1* expression. These results were in accordance with the conclusion we had drawn based on the data from Experiment 1 that these two genes were involved in responses to maternal SCN-derived signals. Interestingly, again in accordance with the Experiment 1 data, exposure of animals fed *ad libitum* to LL had no effect on the rhythmicity of the expression of genes encoding neurotransmitters (*Vip* and *Avp*) (for the cosinor data, see Table 2).

Exposure of pregnant rats in which SCN signaling to fetuses was attenuated due to LL to restricted food availability (LL-RF group) had gene-specific effects within the fetal SCN (see Table 3 for the comparison between the LL-ad lib and LL-RF profiles based on the 2-way ANOVA results). Exposure to RF did not affect the LL-induced suppression of the rhythmicity of *c-fos* and *Nr3c1* expression because their profiles in the LL-RF group were also nonrhythmic. For *c-fos*, 2-way ANOVA detected differences between the LL-ad lib and LL-RF groups only at CT0/24. Interestingly, *Per1* expression, which was nonrhythmic in the CTRL group (Figure 2) as well as in both LL groups (Figure 5), was upregulated in the LL-RF group compared to the LL-ad lib group, whereas the *Per2* expression profile did not differ. We speculate that RF restored the LL-suppressed *Per1* expression. RF exposure had a similar prominent effect on the rhythm in *Vip* expression because amplitude and mesor were increased in the LL-RF group compared to the LL-ad lib group (for the comparison of the amplitudes among the groups, see the cosinor analyses data in Table 2). In contrast, RF exposure had no effect on the rhythmic *Avp* expression, similar to other disruptive stimuli tested in the study. Interestingly, exposure to RF significantly shifted the rhythm of *Nr1d1* expression and induced very shallow rhythms in *Rorα* and *Dbp* expression which were all in synchrony (Figure 5).

Altogether, the results of Experiment 2 demonstrated that exposure of pregnant rats fed *ad libitum* to LL impaired their circadian behavior and the development of their placentas and thus possibly influenced the maternal/fetal barrier. The SCNs of their fetuses responded to the LL-induced attenuation of the maternal rhythmic signals by abolishment of the *c-fos* and *Nr3c1* expression rhythms. Subjecting LL-exposed pregnant rats to feeding/fasting and related activity/rest rhythms had significant gene-specific effects on the fetal SCN; it restored the LL-suppressed expression levels of *Vip* and *Per1* and affected expression of genes related to sensing changes in the cellular metabolic state (*Nr1d1*, *Rorα*, and *Dbp*).

## Discussion

The results of our study provide the first insights into whether and how the fetal SCN clock responds to situations in which the maternal circadian system is challenged via disruption of the environmental LD regime. We demonstrate that exposure of pregnant rats to disruptions resembling situations that humans may experience in their everyday life significantly and selectively impacts gene expression in the fetal SCN. So far, the issue has not been addressed because the impact of maternal chronodisruption on the fetal SCN clock was assessed only after birth, either in newborn pups (El-Hennamy et al., 2008; Nováková et al., 2010) or during later postnatal stages (Mendez et al., 2016).

The SCN of 19-day-old fetuses of the control group, whose mothers were entrained to LD12:12, rhythmically expressed *c-fos*, *Vip*, *Avp* and *Nr3c1*, but the profiles of all other studied genes (*Per1*, *Per2*, *Nr1d1*, *Rorα*, and *Dbp*) failed to meet the requirement for a significant circadian rhythm (for more details, see section “Materials and Methods”). The presence/absence of rhythmicity in expression of these genes as well as acrophases of their rhythmic profiles were in accordance with our previous results (Sládek et al., 2004; Houdek and Sumová, 2014), with the only exception that in the current study, the *Nr1d1* profile did not meet the significance requirements for the circadian rhythm. The abrupt 6 h-phase shift in the LD cycle induced in mothers on gestational day 14 transposed the maternal SCN clock into a transient state during which it gradually became phase-delayed by approximately 1 h a day. Over the course of the next 5 days, the activity/rest ratios calculated for each individual pregnant rat were not aligned with the new LD cycle and in some of the mothers full entrainment was not achieved even on E19, when the fetal SCN were sampled. This transient state affected the gene expression profiles in the SCN of 19-day-old fetuses. Irrespective of whether the genes were expressed rhythmically in controls or not, the expression of most of these genes was robustly suppressed, which included clock (*Per1*, *Per2*, and *Nr1d1*) and clock-controlled (*Dbp*) genes as well as genes involved in sensing various signals, such as *c-fos* and *Nr3c1*. Importantly, *c-fos* was identified as the gene primarily responsible for sensing the phase of the maternal clock because it was expressed rhythmically at E19, and its rhythm was phase-delayed according to the new phase of the maternal SCN clock. In contrast, the transient state of the maternal SCN did not change the rhythmic expression of genes encoding the neurotransmitters *Vip* and *Avp*. The result demonstrates that the rhythm of *Avp* expression, which in the adult SCN is under control of the clock as a clock-controlled gene (Jin et al., 1999), does not follow the phase of *c-fos* after the phase shift, and thus the rhythmic expression profiles of these two genes in the fetal SCN is driven by divergent signals.

To ascertain whether the effects we observed in the fetal SCN due to the transient state were caused by the abolishment or reduction in the rhythmic maternal signals, we exposed the pregnant rats to LL. Exposure to LL affects the ability of maternal SCN to transmit rhythmic signals to the fetuses because previous studies found that under LL, the SCN neuronal activity rhythm was dampened (Lucassen et al., 2016), and the cellular oscillators became mutually desynchronized (Ohta et al., 2005), which had an impact on the production of coherent rhythmic signals driving rhythms at the systemic level. Exposure of pregnant rats to LL starting on E0 caused an initial lengthening of the circadian period of locomotor activity rhythms, which was gradually followed by a complete loss of rhythmicity, as we previously showed for this rat strain (Houdek and Sumová, 2014). The timing of the beginning of arrhythmicity slightly varied among the dams and occurred after approximately 2 weeks under LL, which indicates that between E14 and E19, all but one of the pregnant rats (which was excluded from the study) completely lost behavioral rhythmicity. Therefore, the maternal signals were modulated during the same interval from E14 to E19 as in the previous experiment, in which the pregnant rats were exposed to a phase shift in the LD cycle. Exposure of rats to LL may have a more general impact on the course of pregnancy because it has previously been assigned to be a stressor (Honma and Hiroshige, 1978) and was found to potentially affect sex hormone levels (Takeo et al., 1975). Indeed, we revealed that in the LL-ad lib group, the weights of placentas were significantly reduced compared to those in the CTRL group, which was not caused by a decrease in the dams’ food intake or a reduction in BW gain during pregnancy. Additionally, in our conditions the litter sizes were not affected by LL but the embryo weights were slightly higher than those in the control group. However, another study found that the weights of fetuses or newborn pups were lower in LL-exposed dams (Mendez et al., 2012; Amano et al., 2020).

In line with the results of our phase-shift experiment (as described above), we found that LL completely abolished rhythmic expression of *c-fos* in the fetal SCN but did not affect the rhythms in *Avp* and *Vip* expression. This supports the above proposed scenario of the divergence of the signals driving rhythmicity of *c-fos* and *Avp* expression in the fetal SCN. Additionally, the persistence of the *Vip* and *Avp* rhythms under LL conditions excluded the possibility that the absence of rhythmicity in the expression of genes, which were rhythmic during the LD cycle, was due to a lack of mutual synchrony among otherwise rhythmic SCN clocks in individual fetuses. Therefore, the data are in favor of the explanation that the rhythmic expression of these genes was dependent on presence of maternal signals. This conclusion is in accordance with the hypothesis we formulated earlier about the maternal origin of the rhythmicity detected at this early fetal stage in the rat SCN (Sumová et al., 2012).

Comparisons between the responses of the gene expression profiles in the fetal SCN in both experiments (Figures 2, 5) revealed that they greatly differed. It appeared that the dampening of gene expression we detected under the transition state was not caused simply by the reduction/absence of signals sent from the maternal to the fetal SCN, as occurred under LL. The fetal SCN is thus responding to various maternal pathways that were plausibly specifically modulated due to exposure to these two challenges. However, identifying these mechanisms is problematic because maternal signals to the fetal SCN are complex, interconnected and convergent. They may involve hormonal levels, body temperature as well as activity/sleep and feeding/fasting rhythms (reviewed in Sumová et al., 2012). Regarding the humoral pathways, we may speculate about the involvement of at least two candidate hormones that are controlled by the maternal SCN and might thus play a role in these effects; melatonin, as a messenger of darkness (Davis and Mannion, 1988; Houdek et al., 2015), and recently discovered GCs, as messengers of the active state (Čečmanová et al., 2019). The maternal SCN provides the fetal SCN with combinatory hormonal signaling, i.e., in nocturnal rats, the simultaneously elevated levels of melatonin and GCs are signaling the time when the mother is awake and active. Importantly, these two hormones likely responded to the chronodisruptions tested in our study differently. Under the transient state due to the phase delay in the LD cycle, the maternal SCN gradually delays the timing of the elevation in melatonin levels (Humlová and Illnerová, 1992), which could theoretically drive the delay in the *c-fos* expression profile because the gene expression profile was entrained by melatonin (Houdek et al., 2015). However, the situation is not clear in case of GCs. Expression of their receptor (*Nr3c1*) exhibits circadian variation in the fetal SCN on E19, as we have shown in this study as well as our previous study (Čečmanová et al., 2019). Here, we found that the transient state abolished the circadian rhythm in *Nr3c1* expression, suggesting impaired rhythmicity of the hormonal profile. If this is correct, the impairment of the GC rhythm due to the transient state might play a role in the downregulation of the clock gene expression profiles we detected in the fetal SCN because we have previously shown that GCs may facilitate fetal SCN development (Čečmanová et al., 2019). Therefore, we can speculate that the transient state of the SCN in pregnant rats likely affected the mutual alignment of melatonin and GC signaling to the fetal SCN, which might lead to the deregulation of convergent signaling with functional relevance for the maintenance of gene expression levels. Similar mechanism may be employed in the effect of LL on fetal SCN because it was previously found that LL exposure affects both melatonin and GC levels in pregnant rats (Mendez et al., 2012). The plasma melatonin levels in pregnant rats (Mendez et al., 2012; Houdek et al., 2015) were suppressed to the same extent as in adult males (Wideman and Murphy, 2009; Dauchy et al., 2010). Additionally, we previously confirmed that in the LL-exposed pregnant rats, melatonin injections during E17–E21 (last 5 days of pregnancy) served as a potent synchronizer of the fetal SCN clock as detected in newborn pups (Houdek et al., 2015). Under LL conditions, the amplitude of the corticosterone rhythm was significantly dampened in adult male rats (Claustrat et al., 2008; Park et al., 2013; Tapia-Osorio et al., 2013), but it was rather delayed in pregnant Sprague-Dawley rats at E18 (Mendez et al., 2012). In our Wistar rats, we found significant reduction in the amplitude of the oscillation of *Nr3c1* expression in the fetal SCN in the LL-ad lib group compared with that in the CTRL group, which suggests a significant effect of LL on the maternal GC rhythm. Theoretically, the effect might play a role in abolishment of the *c-fos* rhythm in the LL-ad lib group because we previously demonstrated that dexamethasone application to pregnant rats induced acute responses of *c-fos* expression in the fetal SCN (Čečmanová et al., 2019). Additionally, the fetal adrenal glands were suggested to play a role of a melatonin-sensitive peripheral clock in the mother (Torres-Farfan et al., 2011), providing further potential mechanism of how LL impacts the fetal SCN. Altogether, the LL-induced suppression of melatonin levels supported by a concurrent modulation of the GC rhythm seems to be a plausible mechanism for the effect we observed in the fetal SCN, although the direct connection with the regulation of the studied genes remains unclear.

The exposure of pregnant rats to LL disrupts not only hormonal levels but also other entraining signals, namely, the behavioral activity/sleep and feeding/fasting rhythms as well as the tightly related body temperature rhythm (Eastman and Rechtschaffen, 1983). Re-inducing those rhythms in pregnant rats maintained in LL via temporary restriction of access to food allowed us to ascertain their participation in the effects of LL on the fetal SCN. Previously, we showed that the same protocol was efficient in entraining the clock in newborn rats (Nováková et al., 2010). Here we found that in the fetal SCN the lack of a maternal behavioral rhythm was not involved in abolishment of *c-fos* rhythmicity due to LL exposure, further supporting the abovementioned speculations on the role of hormonal signals in the regulation of genes in the fetal SCN. Unexpectedly, we revealed participation of the maternal behavioral rhythm in maintenance of the *Vip* expression level and the amplitude of its rhythm, suggesting its role in neuronal maturation of the fetal SCN. Apart from this, less pronounced but significant effects on the expression profiles of *Per1* (slight upregulation) and *Nr1d1*, *Rorα*, and *Dbp* (by inducing shallow rhythmicity) were also detected. Interestingly, we previously observed the same effect of RF on *Nr1d1* expression in the adult SCN of rats exposed to LL (Nováková et al., 2011).

Altogether, our study revealed that the fetal SCN responds to complex maternal signals in a gene-specific manner. Importantly, disruption these maternal signals impacts the fetal SCN and affects regulation of genes that are involved in general cellular signaling as well as clock-related mechanisms. It is tempting to speculate that such effects may mediate the noxious impact of prenatal chronodisruption on the development of the SCN.

## Data Availability Statement

The raw data supporting the conclusions of this article will be made available by the authors, without undue reservation.

## Ethics Statement

The animal study was reviewed and approved by the Animal Care and Use Committee of the Institute of Physiology of the Czech Academy of Sciences.

## Author Contributions

VL: design of the work, data acquisition, writing the draft, and final approval of the version to be published. PH: data acquisition and final approval of the version to be published. KL: data acquisition, data analysis, and final approval of the version to be published. AS: conceptualization and data interpretation, writing the manuscript, and final approval of the version to be published. All authors contributed to the article and approved the submitted version.

## Conflict of Interest

The authors declare that the research was conducted in the absence of any commercial or financial relationships that could be construed as a potential conflict of interest.
